# Tinea Cruris

**Published:** 1909-03-13

**Authors:** 


### DERMATOLOGY.

### TINEA CRURIS.

This affection, now known to be a form of ringworm, was first described by Hebra in 1860 under the name of eczema marginatum, and although he did not at first recognise the disease as ringworm, his description has remained classical.

### Hebra's Description.

Hebra taught that it always begins on the inner side of the thigh, first appearing as a red, raised, and circular patch of nearly the size of a sixpence; that the patch itches and is scratched, and thus excoriated; that it then becomes pale in the centre, and spreads at the margin on the thigh and the adjacent part of the scrotum—for it nearly always occurs in males—until it reaches the size of the palm; then other patches appear and extend in circles in a similar manner, and the same events occur in the other groin, until eventually the spreading circles join to form one large festooned margin, extending across the inner side of the thigh, over the buttocks, back along the inner side of the opposite thigh, and across the lower abdomen to complete the circle. At this festooned margin are papules, vesicles, excoriations, and crusts, and within it the skin, though free from these lesions, is more deeply pigmented than the skin outside.

Chiefly on account of the itching, and because of the presence of vesicles and crusts on the red margin, Hebra classed this affection as an eczema. Some few years later, however, other observers demonstrated the presence of fungus in the scales, and the eruption has consequently been transferred from the group of eczemas to that of ringworm. It is now known, too (a fact that was first pointed out by Tilbury Fox), that the disease often occurs in epidemics, and quite recently Sabouraud, in Paris, has succeeded in cultivating a special fungus from the lesions in several epidemics.

A remarkable feature about this particular variety of ringworm is its intractability to treatment, and it is on record that cases have actually persisted for 20 years or more. It is peculiar too that the hairs of the pubic region are never attacked, so that its obstinate nature cannot be attributed to the fungus remaining in the hairs after the skin lesions have been removed. It seems more probable that the persistence of the disease and its tendency to recurrence is due to fresh infection from the patient's clothing.

Large epidemics have been known in schools, colleges, and among the resident staff of hospitals, and these epidemics have sometimes lasted for years. The cases occurring in such epidemics are not always so extensive as those related by Hebra, and the lesions may be limited to the parts about the groin. It is certain that this form of ringworm is not due to any of the many known varieties of fungus which cause scalp, beard, or body ringworms, but whether the particular fungus cultivated by Sabouraud in France will be found to be the cause of the cases in this country remains to be seen.

### Treatment.

With regard to the best form of treatment appropriate to this affection, it has already been said that it is intractable, and even such radical measures as peeling off the skin with iodine paints, or frictions with strong chrysarobin ointment have often failed to produce a permanent cure. Perhaps the most successful of all treatments is that which has been adopted in some of the recent epidemics among hospital residents—namely, that of applying the preliminary dressing for an operation. The parts are first scrubbed with soap and water, then thoroughly soaked with a 1 in 2,000 perchloride of mercury lotion, and then a pad of gauze moistened with the same lotion is applied, and covered with jaconet, and bandaged on. The dressing remains on for several days, being changed at intervals, until the eruption fades. A second course of treatment on the same lines may be successful if recurrence occurs after the first.

